# The shift in sensory eye dominance from short-term monocular deprivation exhibits no dependence on test spatial frequency

**DOI:** 10.1186/s40662-022-00303-4

**Published:** 2022-09-01

**Authors:** Yiya Chen, Yu Mao, Jiawei Zhou, Zhifen He, Robert F. Hess

**Affiliations:** 1 School of Ophthalmology and Optometry and Eye Hospital, and State Key Laboratory of Ophthalmology, Optometry and Vision Science, Wenzhou Medical University, Wenzhou, 325000 Zhejiang China; 2 Department of Ophthalmology and Visual Sciences, McGill Vision Research, McGill University, Montreal, PQ H3A 0G4 Canada

**Keywords:** Monocular deprivation, Binocular combination, Spatial frequency, Visual plasticity, Sensory eye dominance

## Abstract

**Background:**

Studies have shown that short-term monocular deprivation induces a shift in sensory eye dominance in favor of the deprived eye. Yet, how short-term monocular deprivation modulates sensory eye dominance across spatial frequency is not clear. To address this issue, we conducted a study to investigate the dependence of short-term monocular deprivation effect on test spatial frequency.

**Methods:**

Ten healthy young adults (age: 24.7 ± 1.7 years, four males) with normal vision participated. We deprived their dominant eye with a translucent patch for 2.5 h. The interocular contrast ratio (dominant eye/non-dominant eye, i.e., the balance point [BP]), which indicates the contribution that the two eyes make to binocular combination, was measured using a binocular orientation combination task. We assessed if BPs at 0.5, 4 or 6 cycles/degree (c/d) change as a result of monocular deprivation. Different test spatial frequency conditions were conducted on three separate days in a random fashion.

**Results:**

We compared the BPs at 0.5, 4 and 6 c/d before and after monocular deprivation. The BPs were found to be significantly affected by deprivation, where sensory eye dominance shift to the deprived eye (F_1.86, 16.76_ = 33.09, *P* < 0.001). The changes of BP were consistent at 0.5, 4, and 6 c/d spatial frequencies (F_2,18_ = 0.15, *P* = 0.57).

**Conclusion:**

The sensory eye dominance plasticity induced by short-term deprivation is not dependent on test spatial frequency, suggesting it could provide a practical solution for amblyopic therapy that was concerned with the binocular outcome.

**Supplementary Information:**

The online version contains supplementary material available at 10.1186/s40662-022-00303-4.

## Background

Several studies over the past decade have shown that short-term monocular deprivation, from 15 min to 5 h, makes the deprived eye stronger in its contribution to binocular vision in human adults [1–4]. This visual plasticity was first reported by Lunghi and colleagues [1], who showed that after 2.5 h of monocular pattern deprivation, the perceived dominance duration for the deprived eye was longer in a binocular rivalry task where the mean durations of the individual ‘‘deprived eye’’ and ‘‘non-deprived eye’’ percepts were used to calculate the ocular dominance ratios (i.e., shift in sensory eye dominance in favor of the deprived eye). Such effects lasted for about 30 min. Later, Zhou et al. [2] demonstrated that this binocular visual plasticity was also reflected in binocular combination tasks that involved phase, motion and contrast. The underlying mechanism was believed to be a contrast gain reduction of the non-deprived eye and a contrast gain enhancement of the deprived eye resulting from short-term monocular deprivation [5, 6]. Electroencephalographic results [6, 7] reported that the visual evoked potential driven by the deprived eye increased under binocular conditions. Furthermore, a magnetoencephalogram result [5] showed that the activity of the deprived eye was larger while that of the non-deprived eye was reduced when eyes were viewing dichoptically compared with monocular viewing.

The fact that the abovementioned visual plasticity is present in adults and reciprocally modulates the two eyes’ contributions to binocular combination highlights its potential application in patients with binocular disorders. This has now been applied in adults with amblyopia. Amblyopia is a common form of unilateral poor vision which is due to a disruption to normal visual development early in life [8]. Studies have shown that binocular visual deficits in amblyopia are difficult to treat by traditional patching therapy [9–12]. Zhou and colleagues first reported that depriving the amblyopic eye for about two hours was followed by a transient boost of the deprived eye (i.e., amblyopic eye) in binocular viewing [13] and suggested its potential therapeutic application. Subsequently, two groups have found that repeated daily short-term monocular deprivation (of the amblyopic eye) led to the recovery of visual acuity (of the amblyopic eye) as well as a more balanced binocular vision in adult amblyopes [14, 15]. This observation resonates with the idea that sensory eye dominance plasticity may open important new ways for treating adult amblyopia (for review see Basgoze et al. [16], and Castaldi et al. [17]). Moreover, short-term monocular deprivation has been shown to be effective in improving visual performance for retinitis pigmentosa [18] and inducing the same visual plasticity on older adults as younger population [19], and thus further confirms the potential clinical application of such visual plasticity.

Notably, sensory eye dominance changes that occur from short-term monocular deprivation have been mainly studied at low spatial frequency (e.g., 0.3 to 3 c/d) [1, 2, 13] because the phase combination task used was restricted to this low spatial frequency range. The potential therapeutic use of this neuroplastic effect would be strengthened if the rebalancing of binocular vision which it initiates occurs for all spatial frequencies and not just low spatial frequencies. However, if on the other hand, it only rebalances binocular vision for low spatial frequencies, this approach would provide inadequate amblyopia therapy. This is because amblyopia has been reported to have spatial deficits at mid-high spatial frequency [20, 21]. Several recent studies also demonstrated that binocular balance is more disrupted at higher spatial frequencies in amblyopia in binocular rivalry tasks [22] and binocular combination tasks [23]. Chen et al. [11] reported similar spatial frequency dependent binocular imbalance even in clinically cured amblyopia. Therefore, the spatial dependent deficits of amblyopia and treated amblyopia highlight the importance of understanding the test spatial frequency dependence of short-term deprivation in human adults. To the best of our knowledge, few studies have explored such dependence. Binda et al. [24] showed that short-term monocular deprivation selectively boosted the blood oxygen level-dependent (BOLD) response to the deprived eye for high spatial frequency stimuli in primary visual cortex (V1), however this effect was measured with monocular viewing. Sensory eye dominance, an indicator reflecting binocular function, is more relevant to previous studies on plasticity that use short-term monocular deprivation [1, 2]. It is still unclear whether the monocular pattern deprivation with a translucent patch produces different magnitudes of sensory eye dominance shift at different test spatial frequencies.

To address this issue, we conducted a binocular orientation combination task [25] with a previously validated method of adjustment [26] where subjects were asked to adjust the contrast in the dominant eye to obtain a balanced binocular percept to measure the changes of binocular balance at different spatial frequencies after short-term monocular deprivation. This task using the method of adjustment only takes about 3 min for each spatial frequency, and has good test–retest reliability similar to the task with the method of constant stimuli [26]. Here, we chose spatial frequencies of 0.5, 4, and 6 c/d. This is also a spatial frequency range that is typical of binocular deficits in patients with amblyopia [22, 27] and treated amblyopes [11]. We found that the binocular balance at all spatial frequencies measured were affected equally because of short-term monocular deprivation, suggesting the binocular effects of this form of deprivation are not test spatial frequency dependent.

## Methods

### Participants

Ten adults with normal or corrected-to-normal vision (mean age ± SD: 24.7 ± 1.7 years; four males) participated in our study. Although this sample size may appear to be too small to show a robust result, it was chosen based on the data extracted from Fig. 4 of Binda et al. [24]. Power calculation showed that the minimum sample size of eight subjects was adequate to achieve a power greater than 80% in detecting a difference of deprivation effect between two spatial frequencies (− 0.24 ± 0.66 and 0.83 ± 0.74, respectively, for the average changes of V1 BOLD responses to the band-pass noise stimuli with peaks at 0.4 and 2.7 c/d).

If needed, we corrected the refractive errors of observers before collecting data. The inclusion criteria were: (1) best-corrected visual acuity (BCVA) ≥ 20/20; (2) refractive error (spherical equivalent) <  − 6.00 D; (3) stereo acuity (RDS) ≤ 60 arc sec; (4) no strabismus, no history of ocular surgery or trauma. All subjects were naive to the purpose of the study and provided written informed consent. The study was approved by the institutional review boards at Wenzhou Medical University (2019-095-K-89) and  was conducted with adherent to the tenets of the Declaration of Helsinki.

### Apparatus

All experiments were conducted using MATLAB R2016b (The Mathworks, Inc., Natick, MA, USA) with PsychToolBox 3.0.14 extension [28] on a MacBook Pro (13-in., 2017; Apple, Inc., Cupertino, CA, USA). For the binocular orientation combination task, we dichoptically presented stimuli via head-mount goggles that had been Gamma-corrected (GOOVIS, AMOLED display; NED Optics, Shenzhen, China). They had a refresh rate of 60 Hz, a resolution of 2560 × 1600 pixels, and a maximal luminance of 150 cd/m^2^.

### Stimuli and design

**Fig. 1:**
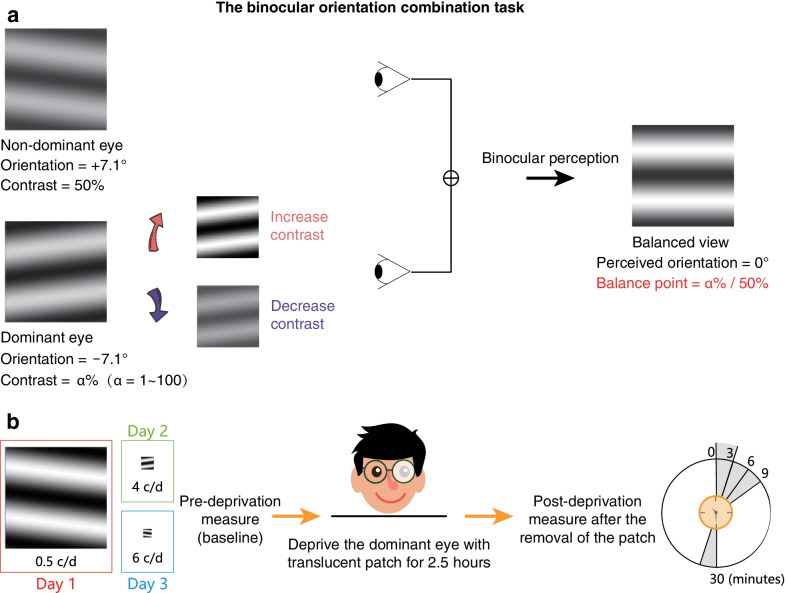
Illustration of the experimental procedure for measuring sensory eye dominance. **a** Binocular orientation combination task. Two horizontal sinusoidal gratings with equal and opposite tilts (± 7.1°) are dichoptically presented to the two eyes. The grating seen by the non-dominant eye has a fixed contrast of 50%, while the grating in the dominant eye has a starting contrast of 1% or 100%. Observers were asked to adjust the contrast in the dominant eye to achieve a balanced binocular viewing, i.e., the binocular perceived orientation = 0°. α% represents the contrast in the dominant eye when the perceived orientation is 0°. Sensory eye dominance (i.e., balance point) is quantified by the interocular ratio (dominant eye/non-dominant eye) that is needed to achieve the balanced binocular viewing. **b** The experimental procedure. We deprive subjects’ dominant eye for 2.5 h, and assess the sensory eye dominance at baseline, 0, 3, 6, 9, 30 min after the completion of the deprivation. Stimuli are set at 0.5, 4 or 6 c/d. Different spatial frequency conditions were conducted on three separate days

The experiment comprised three consecutive stages (Fig. 1b): a pre-deprivation (baseline) measurement of sensory eye balance, a 2.5-h monocular deprivation stage and a post-deprivation measurement of sensory eye balance. For each observer, the dominant eye (tested by the hole-in-the-card test [29]) was chosen for short-term monocular deprivation with a translucent patch, which deprived all forms of information. Observers were free to do any visual work when deprived other than sleeping, while exercise stronger than walking was not permitted in this study to eliminate the potential effect of exercise on results [30, 31]. Binocular orientation combination tasks were used to quantitatively assess the binocular balance at three spatial frequencies (0.5, 4, and 6 c/d; Fig. 1a). When spatial frequency was altered, the number of spatial cycles (i.e., 2 cycles) was held constant. The main outcome measure is the balance point (BP), defined as the interocular contrast ratio (dominant eye/non-dominant eye) in which the two eyes were balanced in binocular combination (i.e., have equal contribution).

In the measurement, we included two orientations of horizontally tilted sinusoidal gratings (for 0.5, 4, and 6 c/d, the size of each grating was 4° × 4°, 0.5° × 0.5°, and 0.33° × 0.33°, respectively), one for each eye, at two different configurations. In the first configuration, the grating shown to the dominant eye had an orientation of + 7.1° counter-clockwise relative to the horizontal position, whereas a counterpart grating shown to the non-dominant eye had that of − 7.1° clockwise relative to the horizontal position. In the second configuration, the grating shown to the non-dominant eye had an orientation of + 7.1° counter-clockwise relative to the horizontal position, whereas a counterpart grating shown to the dominant eye had that of − 7.1° clockwise relative to the horizontal position. As a result, the total difference of orientation between the eyes was 14.2°. The base contrast (i.e., Michelson contrast) of the grating shown to the non-dominant eye was fixed at 50%. In different trials, the starting contrast of the grating shown to the dominant eye was either at 100% or 1%. The trials based on the different orientation configurations and starting contrasts were randomized. Observers were asked to adjust the contrast of the grating presented to the dominant eye until they perceived a horizontal orientation of cyclopean sine-wave grating (i.e., perceived orientation = 0°). Using this approach, observers’ sensory eye dominance (BP) was tested before the deprivation and at 0’, 3’, 6’, 9’ and 30’ after the completion of the 2.5 h of monocular deprivation. We tested each observer’s BP twice at baseline for each experiment and chose to analyze the second measurement. These two measurements were made to confirm that all subjects were familiar with the task with a stable performance before monocular deprivation. We took the second measurement for analysis simply because the first one would be more considered as a practice. If the deprived eye became stronger, the BP would decrease (i.e., the change of BP became negative), otherwise, the BP would increase (i.e., the change of BP became positive). Proper demonstrations were provided with detailed introduction and practice trials to ensure observers had understood the task. Different spatial frequency conditions were conducted in a random order on separate days for different observers. The period between two deprivation sessions was at least one day (i.e., more than 24 h), the average period was 17.90 ± 26.88 days.

### Procedure

As was the case in our previous study [26], there was an alignment phase and a test phase in a typical trial of the binocular orientation combination task. During the alignment phase, subjects were asked to align the dichoptic presented crosses with fusion surrounding frame and diagonal bars in the two eyes. The alignment phase ensured all subjects were correctly aligned throughout the block. The coordinates of the two crosses were then used to present the stimuli in the following test phase. Next, a blank screen (comprised of a surrounding frame and diagonal bars in each eye to facilitate fusion) was displayed for 500 ms. This followed the test phase, during which a horizontal sinusoidal grating with a differing tilt was shown to each eye (Fig. 1a). The contrast of the grating shown to the non-dominant eye was maintained at 50%. The starting contrast was either at 100% or 1% for the dominant eye. Observers were asked to adjust the contrast of the grating presented to the dominant eye in either 5% (fine) or 10% (coarse regulation) steps with ‘up’ or ‘down’ key, until they perceived a horizontal orientation of cyclopean sine-wave grating. The gratings were shown until subjects completed the task by pressing the ‘space’ key. The next trial then started automatically after the subjects’ responses. Each orientation configuration was repeated 4 times. Therefore, there were 16 trials (2 orientation configurations × 2 starting contrasts × 4 repetitions) conducted in one test session for each spatial frequency. BP was calculated as the average interocular contrast ratio of 16 trials.

### Statistical analysis

We used SPSS v.23.0 (IBM Corporation, Armonk, NY, USA) to perform a two-way repeated-measures analysis of variance (ANOVA) with sphericity correction to evaluate the effect of time after deprivation and spatial frequency on binocular balance. Then, we computed area under the curve (AUC):

AUC = 3 min × (|change of BP at 0’| +|change of BP at 3’|)/2 + 3 min × (|change of BP at 3’| +|change of BP at 6’|)/2 + 3 min × (|change of BP at 6’| +|change of BP at 9’|)/2

as a unit that represents the whole deprivation effect of the first 9 min across spatial frequency. We used a one-way repeated-measures ANOVA with sphericity correction to evaluate the effects of spatial frequency or time session after deprivation on binocular balance, respectively. Differences in means were considered statistically significant when *P* < 0.05. In addition, we used the JASP software [32] to perform a Bayesian repeated measures ANOVA to calculate the lgBF to quantify the evidence for or against the null hypothesis, with |lgBF|> 0.5 indicating substantial evidence for (negative lgBF) or against (positive lgBF) the null hypothesis [33].

## Results

**Fig. 2:**
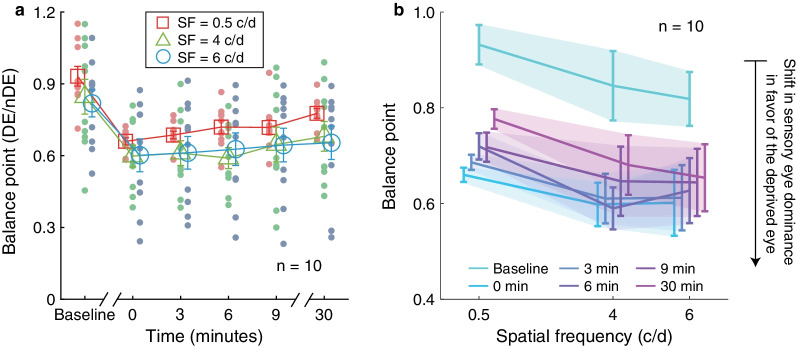
The effect of 2.5 h of monocular deprivation on sensory eye dominance at 0.5, 4, and 6 c/d. **a** The balance point (dominant eye/non-dominant eye, DE/nDE) as a function of various time points before and after deprivation at different spatial frequencies. Open symbols denote the average balance points across ten subjects; red square symbol represents the result of spatial frequency at 0.5 c/d; green triangle symbol represents the result of spatial frequency at 4 c/d; blue circle symbol represents the result of spatial frequency at 6 c/d. Error bars indicate standard errors. **b** The balance point as a function of spatial frequency. Each line denotes the average balance point across ten subjects at one-time session (i.e., baseline, 0, 3, 6, 9, or 30 min after the finish of deprivation) with a jitter. Error bars signify standard errors

We measured the interocular ratio (dominant eye/non-dominant eye) that is needed to achieve a balanced binocular percept (i.e., BP), before deprivation and at 0’, 3’, 6’, 9’ and 30’ after the completion of the 2.5 h of monocular deprivation. Firstly, we conducted a Shapiro-Wilks test to check for normality assumption of the BP at each pre- and post-measurement session (all *P* > 0.05). Then, a Pearson correlation analysis suggested that the binocular orientation combination task we used had a good test–retest reliability which showed a strong correlation between two test sessions at baseline for all spatial frequency conditions (r = 0.941, 0.981, 0.984, respectively, for 0.5, 4, 6 c/d, all *P* < 0.001). Following that, we calculated the change of the BP after the completion of short-term monocular deprivation separately at three test spatial frequencies. Figure 2a shows the individual and average group BP before and after deprivation of the dominant eye for 0.5, 4, 6 c/d spatial frequencies as red square symbols, green triangle symbols, and blue circle symbols, respectively. Before deprivation, a binocular balance was reached when the dominant eye's contrast was 46.59% ± 0.29%, 42.30% ± 0.51%, and 40.92% ± 0.40%, respectively, for 0.5, 4, and 6 c/d spatial frequency; but after deprivation only 33.00% ± 0.11%, 29.91% ± 0.32%, and 30.07% ± 0.49% contrast were needed to balance the 50% contrast shown to the non-dominant eye at 0’ post-measurement session.

We performed a two-way repeated-measures ANOVA, with the time session (six levels) and spatial frequency (three levels) selected as within-subject factors, to assess the short-term monocular deprivation effect on the BP across test spatial frequencies. The results showed that BPs were significantly different across time sessions for all spatial frequencies we tested (Time session: F_1.86, 16.76_ = 33.09, *P* < 0.001, Partial η^2^ = 0.786, lgBF = 18.02; Time session × Spatial frequency: F_24.76, 42.84_ = 0.93, *P* = 0.509, Partial η^2^ = 0.094, lgBF =  − 3.99). Post-hoc analysis with Bonferroni correction showed that there was a significant difference between the BP at each post-measure session and baseline at all three test spatial frequencies (all *P* < 0.01). These results indicated that the deprived eye was strengthened at all test spatial frequencies we measured after the deprivation of the dominant eye, and such a deprivation effect lasted for at least 30 min for all test spatial frequencies. No significant difference of BP was found among three test spatial frequencies (F_2, 18_ = 1.24, *P* = 0.312, Partial η^2^ = 0.121, lgBF = 2.55), while positive lgBF suggested that we could not draw a conclusion as to whether test spatial frequency influences the deprivation effect. Figure 2b plotted the BP as a function of spatial frequency. We computed the slope of the BP as a function of spatial frequency, and the slopes at all time sessions were not significantly different from 0 (one-sample t-test, two-tailed, all *P* > 0.05). We subsequently compared the slopes across time sessions. One-way repeated ANOVA reported that the slope did not change significantly: F_2.63, 23.63_ = 1.133, *P* = 0.357, Partial η^2^ = 0.112, lgBF =  − 1.38, suggesting a comparable change of BP across test spatial frequencies. Since BP only reflects the dominant contribution of the deprived eye to the binocular combination, which may be larger than 1 as seen in Fig. 2a, to assess whether the monocular pattern deprivation produces different magnitudes of binocular balance change at different test spatial frequencies, we calculated the |logBP| (i.e., an indicator of binocular imbalance, where the larger the |log BP|, the more binocular imbalance), and found similar results as BP (see Additional file 1).

**Fig. 3:**
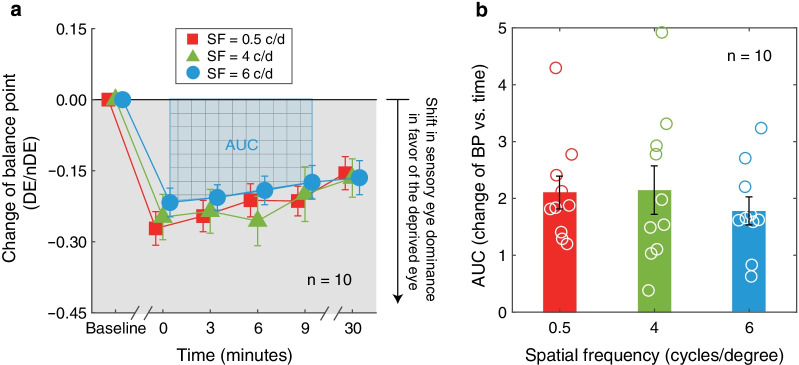
The effect of 2.5 h of monocular deprivation on sensory eye dominance revealed by the changes of balance point (BP) at 0.5, 4, and 6 c/d. **a** The changes of the BPin the function of various time points before and after deprivation at different spatial frequencies. Filled symbols denote the average BPchanges across ten subjects with a jitter; red square symbol represents the result of spatial frequency at 0.5 c/d; green triangle symbol represents the result of spatial frequency at 4 c/d; blue circle symbol represents the result of spatial frequency at 6 c/d. Error bars denote standard errors. More negative indicates that the deprived eye was getting stronger. **b** The area under the curve (AUC) was calculated from (**a**). The values were averaged across ten subjects. Each circle represents the AUC of each subject. Error bars denote standard errors

To further compare the deprivation effects at different spatial frequencies directly, we calculated the changes of BP by subtracting the baseline value from the post-test value. The average changes of BP after deprivation of the dominant eye for 0.5, 4, 6 c/d spatial frequencies are plotted in Fig. 3a as red square symbols, green triangle symbols, and blue circle symbols, respectively. As shown, the BP changed in a more negative direction for all the three spatial frequency conditions. One interesting result, which is also shown in Fig. 3a, was that the deprivation effects were consistent across the three spatial frequencies. A two-way repeated-measures ANOVA with the post-measure session (five levels) and spatial frequency (three levels) selected as within-subject factors, showed that the BP significantly varied from 0’ to 30’ post-measure sessions: F_4, 36_ = 9.27, *P* < 0.001, Partial η^2^ = 0.507, lgBF = 3.63, and the deprivation effect decreased at 30’ shown by post-hoc Bonferroni analysis (change of BP at 0’, 3’, 6’ versus that at 30’, all *P* < 0.02); while the magnitude of the change of BP was not significantly different among the three conditions (i.e., 0.5 c/d versus 4 c/d versus 6 c/d): F_2,18_ = 0.15, *P* = 0.57, Partial η^2^ = 0.059, lgBF = − 0.90; the interaction between spatial frequency (i.e., 0.5 c/d versus 4 c/d versus 6 c/d) and the post-measure sessions (i.e., from 0’ to 30’) was not significant: F_8,72_ = 1.07, *P* = 0.40, Partial η^2^ = 0.106, lgBF =  − 2.88.

**Fig. 4:**
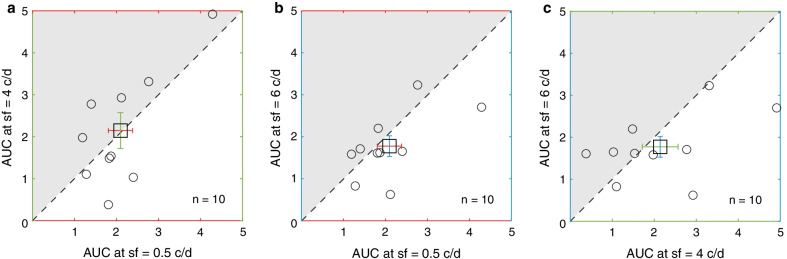
The correlation between area under the curve (AUC) at 0.5 and 4 c/d (**a**), 0.5 and 6 c/d (**b**), 4 and 6 c/d (**c**) spatial frequency. Opened square symbol represents the average AUC across ten subjects. Each circle represents the AUC of each subject. Error bars denote standard errors

To further illustrate the similarity among the three conditions, the area under the curve (AUC) was calculated between the change of BP plots (y-axis) and four post-measure sessions (0, 3, 6, 9 min in x-axis) for each observer (see Fig. 3b). A repeated-measures ANOVA still showed that there was no significant difference of AUC among three spatial frequencies: F_2, 18_ = 0.83, *P* = 0.45, Partial η^2^ = 0.085, lgBF =  − 1.38. What’s more, based on the effect size and the variance in our samples at 0.5, 4, and 6 c/d spatial frequencies (mean ± SD: 2.10 ± 0.91 for 0.5 c/d, 2.14 ± 1.35 for 4 c/d, and 1.78 ± 0.78 for 6 c/d), we found that the sample size would have to be at least 6136, 139, and 75 to reach an 95% power and two-tailed significance level at α = 0.05, respectively, between 0.5 and 4 c/d, 4 and 6 c/d, 0.5 and 6 c/d. The result in turn indicated that the difference between different spatial frequencies was not clinically meaningful. Pearson correlation analysis (see Fig. 4) showed that, there was a significant positive correlation across observers between AUC at 0.5 and 4 c/d spatial frequency (r = 0.715, *P* = 0.020), while no significant correlation was found between AUC at 0.5 and 6 c/d, or 4 and 6 c/d spatial frequency (r = 0.608, *P* = 0.062*,* r = 0.485, *P* = 0.156).

## Discussion

To explore how monocular deprivation influences the binocular combination at different spatial frequencies, we conducted a simplified binocular orientation combination task at low to relatively high spatial frequencies (i.e., 0.5, 4 and 6 c/d). Our results show that short-term monocular deprivation could significantly shift the sensory eye dominance in favor of the deprived eye at both low and high spatial frequencies. We also show that such a deprivation effect does not depend on test spatial frequency.

The change of BP in our study was comparable with that of previous studies which also quantify the sensory eye balance using interocular contrast ratio to assess the short-term effect of depriving the dominant eye [4, 34]. In particular, the contrast of the dominant eye that was needed to balance the non-dominant eye, decreased by 24.5% from base contrast after monocular deprivation. This is similar to a previous study that directly measured BP changes after monocular deprivation. Min et al. [4] reported that the required contrast of the dominant eye to achieve a binocular balance for binocular phase combination decreased by about 20% of the base contrast presented to the non-dominant eye after short-term monocular deprivation. We did not find the spatial frequency dependence of sensory eye dominance in normal adults, as the slope of BP or |logBP| as a function of spatial frequency was not significantly different from 0, which was consistent across different time sessions. Although this was not the main aim of the study, we still want to point out that only normal subjects (n = 10) were tested in our study. These observers were expected to have balanced binocular percept. It is thus not surprising to find a stable binocular balance across spatial frequency in normal observers. In fact, a flat sensory eye dominance vs. spatial frequency curve in normal adults has also been found in previous studies measured with binocular combination [11, 23, 26] and binocular rivalry [22]. However, sensory eye dominance of different types of subjects could differ on the character of spatial frequency dependence, such as amblyopia [23] and myopic anisometropia [35], which is worthy of further study in the future. In addition, significant correlations of deprivation effect were found between some but not all test spatial frequencies in this study. However, considering the large individual variations, a larger sample size would be necessary to make an exclusive conclusion with strong power for the correlation analysis.

To prevent binocular rivalry, we used a similar design as Wang et al. [25] to measure the binocular balance at different test spatial frequencies. In particular, the gratings were fixed at 2 cycles and the interocular orientation difference was fixed at 14.2 degrees. This means that the physical size of the gratings is smaller as the spatial frequency increases. Such a design would enable a fixed interocular phase difference of 90 degrees at the edge of the gratings when measuring binocular balance at different spatial frequencies. Previously, Ding and Sperling [36] and Huang et al. [37] have shown that both normal adults and adult amblyopes were able to fuse an interocular phase difference of up to 135 degrees (i.e., no binocular rivalry). For the oriented gratings that we used here, the interocular phase difference from the left edge to the right edge was within 90 degrees, which would be within the range of binocular phase combination based on Ding and Sperling [36] and Huang et al. [37]. On the other hand, Spiegel et al. [38] and Yehezkel et al. [39] presented data showing that human adults were able to fuse two slightly differently orientated monocular gratings when the interocular orientation difference was less than 20 degrees. Therefore, taking these two previous findings into consideration, we are confident that our task was a measure of binocular combination and did not involve rivalry. In our binocular orientation combination task, one of the dichoptic pair of gratings was shown continuously to the non-dominant eye with a contrast of 50%, and the other one which was adjustable (no time constraint) was presented to the dominant eye. Adaptation or negative afterimages may have affected binocular perception during the measurements. However, such effect exists at all test sessions both before and after deprivation. Thus, adaptation or negative afterimages, even if existed, could not account for our results and conclusion.

Studies have suggested that exercise and attentional selection during the deprivation period can modulate the effect of short-term monocular deprivation [15, 30, 40, 41]. In our study, although we did not strictly constrain this, all the subjects were doing visual work such as using mobile phones or computers during deprivation, with no exercise stronger than walking within the lab. Therefore, such limited activity during the deprivation period would not be expected to have a significant impact on the conclusions of our study.

Here, we only tested binocular balance at 0.5, 4, and 6 c/d spatial frequency. The sensory eye dominance plasticity induced by short-term monocular deprivation was previously only found at low spatial frequencies due to measurement limitations. The binocular orientation combination task has been shown to have a good reliability to be able to extend binocular combination measurements to higher spatial frequencies than previously measured. This new approach allows us to assess the deprivation effect at higher spatial frequencies [25, 26]. However, as we show in Fig. 2, the results are more variable at higher spatial frequencies, which also has been reported in previous studies [11, 23, 25]. Such variation leads to an upper limit to the spatial frequency range that can be tested. This is true for our task as it is for other tasks [22]. In addition, the range of test spatial frequency that can be tested is the range that has reflected the binocular deficit exhibited by patients with amblyopia [22, 27] and treated amblyopia [11]. Stimuli with spatial frequency higher than 6 c/d are less visible, and sensory eye dominance at higher spatial frequency is difficult to be measured due to the limitation of the measurements and interocular suppression in amblyopia [21, 23]. This suppression could not simply be accounted for by the poor visual perception of the amblyopic eye, and treated amblyopes with normal monocular visual acuity still show strong binocular imbalance in the spatial frequencies we tested (0.5 to 6 c/d) [11]. Short-term monocular deprivation has been found to modify sensory eye dominance through adjusting interocular suppression [42]. Therefore, we expected the effectiveness of monocular deprivation in higher spatial frequency so long as it can be detected.

It is important to make a distinction between the spatial frequency selectivity of the induction of the neuroplastic change and the spatial frequency selectivity of the resultant neuroplastic change itself. Such distinction has been shown for the color selectivity of the deprivation effect [43]. Zhou et al. suggested that an unselective monocular deprivation affected achromatic and chromatic contrast responses in a comparable way, while depriving color information plays only a weak role in the induction of sensory eye dominance changes [43]. It has been previously shown that the deprivation of image contrast at high spatial frequencies is important for producing sensory eye dominance changes [44]. What we have shown here is that the monocular pattern deprivation with a translucent patch produces similar magnitudes of sensory eye dominance shift at different test spatial frequencies. Spatial frequency has been shown to be represented continuously across the primary visual cortex (i.e., V1) and that there are multiple spatial frequency processing cells at each location, though little apparent evidence for a columnar organization in adult cats and prosimian bush baby [45, 46]. The binocular orientation combination paradigm that we used here represents a basic binocular function that is likely to be processed mainly by cells in the primary visual cortex that have ocular dominance preference [47]. The deprivation effect on binocular combination has broad test spatial frequency selectivity, which is not unexpected given the fact that multiple spatial frequencies are represented in each ocular dominance column in V1.

Few studies have explored that there is a selective test spatial frequency response of short-term monocular deprivation. Binda et al. [24] by using 7 T functional magnetic resonance imaging, showed that short-term monocular deprivation boosts the BOLD response to the deprived eye, which presented a selectivity for the high spatial frequencies in V1 plasticity. Note that in their study, a broad band-pass noise stimulus was used monocularly to measure the spatial frequency selectivity of each eye. While in our study, with a binocular combination task, we found comparable deprivation effects on binocular combination for different spatial frequencies. The present findings are consistent with a previous report from Spiegel et al. [48], in which they examined how the relationship between suppression, fusion and diplopia changes as a result of short-term monocular deprivation using a binocular task with blurred tilted edges at different levels of blur/spatial scale. They found that there was a change in the inhibitory interaction for fusible stimuli, but such a change did not depend on stimulus spatial scale. Binda et al. hypothesized that such deprivation effect involved the parvocellular pathway to a greater extent [24]. The present results suggest that the neuroplastic changes affect achromatic spatial frequencies ranging from 0.5 to 6 c/d. Parvocellular cells are known to respond selectively to achromatic stimuli of high spatial frequencies and to chromatic stimuli of low to medium spatial frequency [49, 50]. In a previous study, we showed that chromatic stimuli were not selectively affected by monocular deprivation nor were they more effective in inducing deprivation effects for binocular vision [43]. Here, we show that the deprivation effects are not any greater at higher spatial frequencies. Taken together, our findings and the literature to date do not support the idea that monocular deprivation is selective for parvocellular function. Given that we did not test at a range of higher spatial frequencies, it is not possible from our study to determine whether there was greater parvocellular involvement underpinning our results.

Different monocular or binocular tasks may lead to task-specific results since a specific psychophysical task may target a distinct level of spatial processing in the cortex. For instance, Zhou et al. reported comparable achromatic and chromatic sensory eye dominance changes in binocular phase combination [43], while Lunghi et al. found an enhancement bias towards color in binocular rivalry [51]. Pairing exercise with monocular patching has been shown to strengthen the short-term monocular deprivation effect that is quantified by a binocular rivalry task [31] (but see Finn et al. [52] and Virathone et al. [30]), while no such enhancement effect was found in measuring with a binocular combination task [53]. Furthermore, Bai et al. [54] reported that depriving the Fourier phase information of one eye boosted the deprived eye’s dominance during interocular competition but not interocular phase combination. Our conclusions were obtained using a binocular combination task and are relevant to the stage of visual processing that such a task reflects.

Zhang et al. [55] reported that 4 h of binocular orientation deprivation temporarily enhanced normal adults’ contrast sensitivity at the deprived orientation, which was proposed to be an outcome of releasing visual adaptation. Contrast adaptation in the cortex is known to be selective for both orientation and spatial frequency [56]. This binocular deprivation is very different from the monocular deprivation reported here as they by definition occur at different stages of cortical processing. Monocular deprivation causes shifts in sensory eye dominance, binocular deprivation causes shifts in contrast sensitivity. Binocular deprivation is tuned for orientation [55], monocular deprivation is neither tuned for orientation [57] nor spatial frequency as illustrated in the current study. This suggests that such binocular and monocular short-term deprivation involve different mechanisms at different stages of cortical processing.

Short-term monocular deprivation has been shown to improve the monocular and binocular function of amblyopes [13–15], as well as modulating visual plasticity in older adults [19]. However, the clinical application of short-term monocular deprivation is still in its infancy. Previous studies merely reported such deprivation effects at low spatial frequency [1, 2, 13]. No matter how strong the effect is, if short-term monocular deprivation only affects binocular balance at the low spatial frequency, it would be less useful for the treatment of amblyopic patients who have been found to have more profound binocular imbalances at mid-high spatial frequencies [11, 22, 23]. Our study confirms the effectiveness of short-term monocular deprivation to induce sensory eye dominance plasticity in normals at relatively high spatial frequencies, a prerequisite for its clinical usefulness.

## Conclusions

The sensory eye dominance could be significantly shifted in favor of the deprived eye by short-term monocular deprivation at all spatial frequencies measured, and such plasticity was not dependent on test spatial frequency. Therefore, we do confirm the efficiency of short-term monocular deprivation effect at low to high spatial frequencies. This could help to strengthen the empirical foundation for a new therapy in adults with binocular disorders.

## Supplementary Information

**Additional file 1.** The effect of 2.5 h of monocular deprivation on |logBP| at 0.5, 4, and 6 c/d.

## Data Availability

The datasets used and/or analyzed during the current study are available from the corresponding author on reasonable request.
