# Supplementary material for: The shift in sensory eye dominance from short-term monocular deprivation exhibits no dependence on test spatial frequency
Source: Eye Vis (Lond). 2022 Sep 1;9:32. doi: 10.1186/s40662-022-00303-4 (PMC9434876; doi:10.1186/s40662-022-00303-4)

**Additional material**

Indeed, the effect of spatial frequency on the pre-deprivation balance point (BP) was evident (Figure 2b). However, we did not find any significant correlation of BPs across different test spatial frequencies either at baseline or 0’ post-measurement session (Pearson correlation analysis, all *P > 0.05*). It should be noted that BP only reflects the dominant contribution of the deprived eye to the binocular combination, which may be larger than 1 as seen in Figure 2a. Previous studies showed significant spatial frequency dependent of eye dominance in adult amblyopes and treated amblyopes, while such dependence was not significant in normals as our study shows [11, 22, 23].

To determine whether the monocular pattern deprivation produce different extents of binocular balance change at different test spatial frequencies, we also calculated the |logBP| (i.e., an indicator of binocular imbalance, where the larger the |logBP|, the more binocular imbalance). We separately plotted |log BP| at pre- and post-measure sessions as a function of spatial frequency (see Figure S1). A two-way repeated-measures ANOVA showed that the binocular imbalance significantly varied from baseline to post-measure sessions: F_1.99, 17.90_= 22.98, *P < 0.001*, Partial η^2^ = 0.719, lgBF = 8.007*,* while no significant difference across the three test spatial frequencies (F_2, 18_= 1.69, *P = 0.231*, Partial η^2^ = 0.158, lgBF = 5.634) and no significant interaction (F_4.49, 40.45_= 0.87, *P = 0.503*, Partial η^2^ = 0.088, lgBF = −4.024) were shown. No significant correlation of |logBP| was found across different test spatial frequencies either at baseline or 0’ post-measurement session (Pearson’s correlation analysis, except 4 c/d and 6 c/d at 0’ post-measurement session: r = 0.689, *P = 0.028*, other *P > 0.05*). We further computed the slope of |log BP| as a function of spatial frequency, and the slopes at all time points were not significantly different from 0 (One-sample t-test, two-tailed, all *P > 0.05*). We subsequently compared the slopes across time points. One-way repeated ANOVA reported that the slope did not significantly change: F_5, 45_ = 1.115, *P* = 0.366, Partial η^2^ = 0.110, lgBF = −1.407, suggesting a comparable change of |log BP| across test spatial frequencies.

**Figure S1.** The binocular imbalance, that is, the absolute value of the balance point (BP) in log scale (|logBP|), at various time points before and after deprivation. The |logBP| as a function of spatial frequency. Each line denotes the average |logBP| across ten subjects at one time point (i.e., baseline, 0, 3, 6, 9, or 30 minutes after the finish of deprivation). Error bars show standard errors.


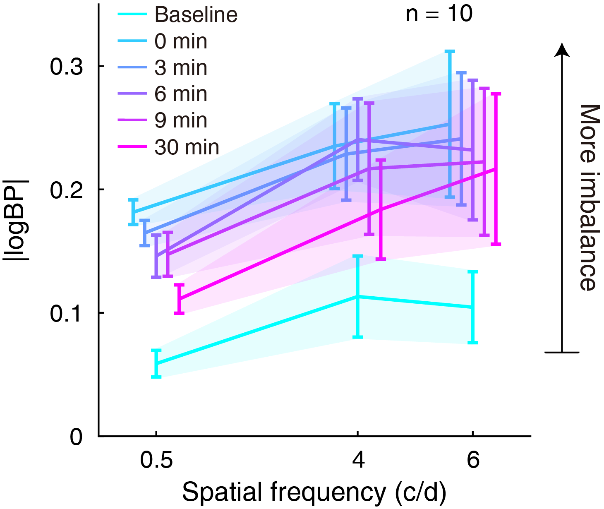

Supplement: Supplementary file 1 — Additional file 1. The effect of 2.5 h of monocular deprivation on |logBP| at 0.5, 4, and 6 c/d. [file 40662_2022_303_MOESM1_ESM.docx]
